# Supplementary material for: Multicolor lifetime imaging and its application to HIV-1 uptake
Source: Nat Commun. 2023 Aug 17;14:4994. doi: 10.1038/s41467-023-40731-x (PMC10435470; doi:10.1038/s41467-023-40731-x)
Supplement: Supplementary file 1 — Supplementary Information [file 41467_2023_40731_MOESM1_ESM.pdf]

Supplementary information for

Multicolor Lifetime Imaging and its Application to HIV-1 Uptake

Tobias Starling[1], Irene Carlon-Andres[1], Maro Iliopoulou[2,3], Benedikt Kraemer[4], Maria Loidolt-Krueger[4], David J. Williamson[1], Sergi Padilla-Parra[1,2,5*]

[1]Department of Infectious Diseases, King's College London, Faculty of Life Sciences & Medicine, London, United Kingdom.
[2]Division of Structural Biology, Wellcome Centre for Human Genetics, University of Oxford. Oxford, United Kingdom
[3]Current Address: [2]Randall Division of Cell and Molecular Biophysics and Department of Physics, King's College London, London, United Kingdom.
[4] PicoQuant GmbH, Rudower Chaussee 29 (IGZ), 12489, Berlin, Germany.
[5]Randall Division of Cell and Molecular Biophysics, King's College London, London, United Kingdom.

* Correspondence: sergio.padilla_parra@kcl.ac.uk

a

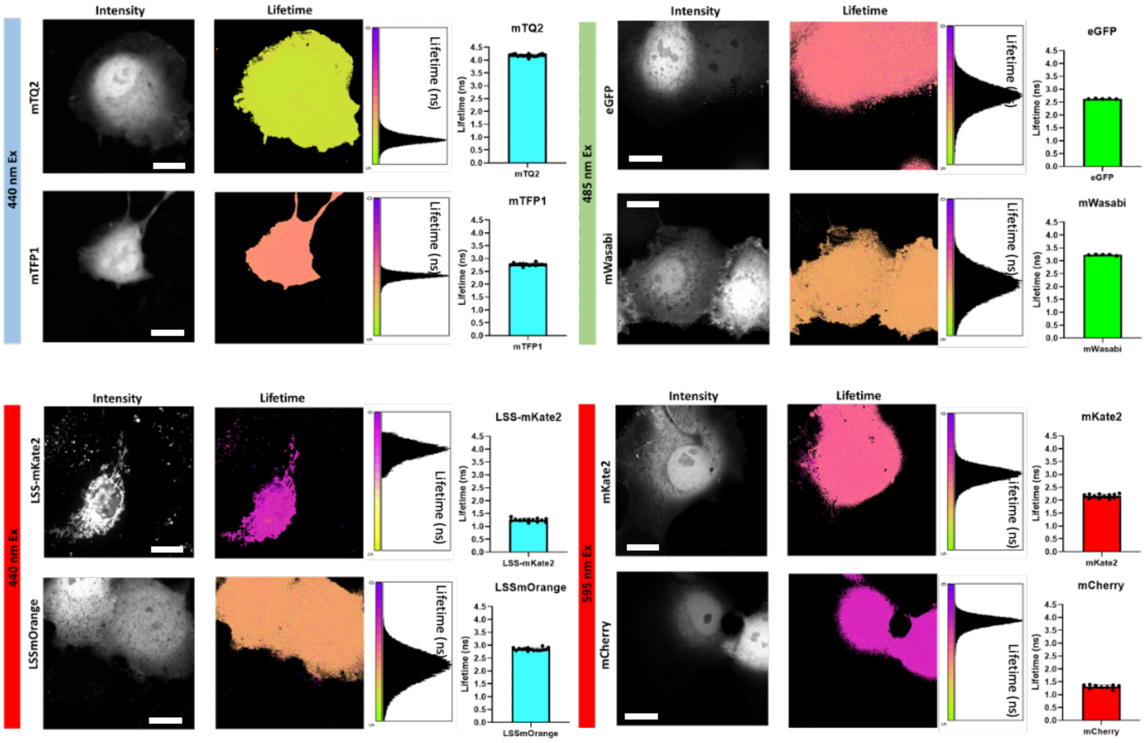
Intensity
Lifetime
440 nm Ex
mTQ2
mTFP1
Lifetime (ns)
mTQ2
mTFP1
485 nm Ex
eGFP
mWasabi
LSS-mKate2
LSSmOrange
595 nm Ex
mKate2
mCherry

b

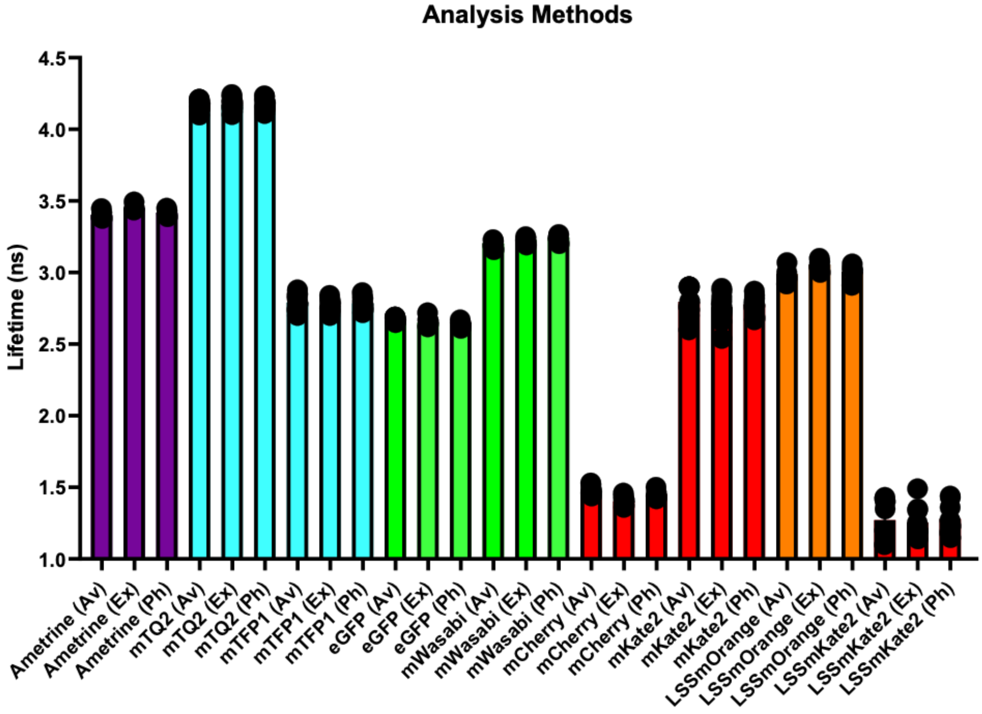
Analysis Methods
Lifetime (ns)
4.5
4.0
3.5
3.0
2.5
2.0
1.5
1.0
Ametrine (Av)
Ametrine (Ex)
Ametrine (Ph)
mTQ2 (Av)
mTQ2 (Ex)
mTQ2 (Ph)
mTFP1 (Av)
mTFP1 (Ex)
mTFP1 (Ph)
eGFP (Av)
eGFP (Ex)
eGFP (Ph)
mWasabi (Av)
mWasabi (Ex)
mWasabi (Ph)
mCherry (Av)
mCherry (Ex)
mCherry (Ph)
mKate2 (Av)
mKate2 (Ex)
mKate2 (Ph)
LSSmOrange (Av)
LSSmOrange (Ex)
LSSmOrange (Ph)
LSSmKate2 (Av)
LSSmKate2 (Ex)
LSSmKate2 (Ph)

Supplementary Figure 1. Fluorescent Lifetime determination for each fluorescent protein when expressed in live cells at physiological conditions. (a) A sample of eight different live cells expressing one fluorescent protein species are depicted. Intensity micrographs (Left row) are depicted together with lifetime images (right micrograph) pixel lifetime histograms and bar charts for the average lifetime of each specific fluorescent protein. The lifetimes in each bar chart are: for mTQ2 (4.2±0.1nanosecods (ns) n = 15), for mTFP1 (2.8±0.1ns n = 14), for eGFP (2.6±0.0ns n = 5), for mWasabi (3.2±0.01ns n = 5), for mito-LSSmKate2 (1.2±0.1ns n = 12), for LSSmOrange (2.8±0.1ns n = 15), for mKate2 (2.2±0.1ns n = 12) and for mCherry (1.3±0.1ns n = 9). n denotes number of viruses examined over 3 independent experiments. The blue, green and red channels are shown. Scale bar 5 µm. Cells expressing Mito-LSSmKate2 were tested for lifetime measurement and expression, the other cells were expressing the corresponding fluorescent proteins freely diffusing in the cytosol. (b) Bar diagram showing the calculation of lifetimes for the nine fluorescent proteins chosen for multicolor FLIM employing the average lifetime (AV), the fitting approach (EX) and the Phasor Plot (Ph). The values for each fluorescent protein employing the three approaches were: for Ametrine (3.4±0.0ns n = 5 (Average Lifetime), 3.45±0.0ns n = 5 (Exponential fit) and 3.4±0.0ns n = 5 (Phasor)) for mTQ2 (4.2±0.0ns n = 15 (Average Lifetime), 4.2±0.0ns n = 15 (Exponential fit) and 4.2±0.0ns n = 15 (Phasor)) for mTFP1 (2.8±0.0ns n = 12 (Average Lifetime), 2.8±0.0ns n = 12 (Exponential fit) and 2.8±0.0ns n = 12 (Phasor)) for eGFP (2.7±0.0ns n = 15 (Average Lifetime), 2.7±0.0ns n = 15 (Exponential fit) and 2.7±0.0ns n = 15 (Phasor)) for mWasabi (3.2±0.0ns n = 5 (Average Lifetime), 3.2±0.0ns n = 5 (Exponential fit) and 3.2±0.0ns n = 5 (Phasor)) for mCherry (1.5±0.0ns n = 10 (Average Lifetime), 1.4±0.0ns n = 10 (Exponential fit) and 1.4±0.0ns n = 10 (Phasor)), for mKate2 (2.8±0.0ns n = 13 (Average Lifetime), 2.8±0.0ns n = 13 (Exponential fit) and 2.8±0.0ns n = 13 (Phasor)), for LSSmOrange (3.0±0.0ns n = 5 (Average Lifetime), 3.1±0.0ns n = 5 (Exponential fit) and 3.0±0.0ns n = 5 (Phasor)) and for LSSmKate2 (1.3±0.0ns n = 9 (Average Lifetime), 1.3±0.0ns n = 9 (Exponential fit) and 1.3±0.0ns n = 9 (Phasor)). n denotes number of viruses examined over 3 independent experiments. Data are presented as mean values +/- SD" as appropriate. Source data are provided with this paper.

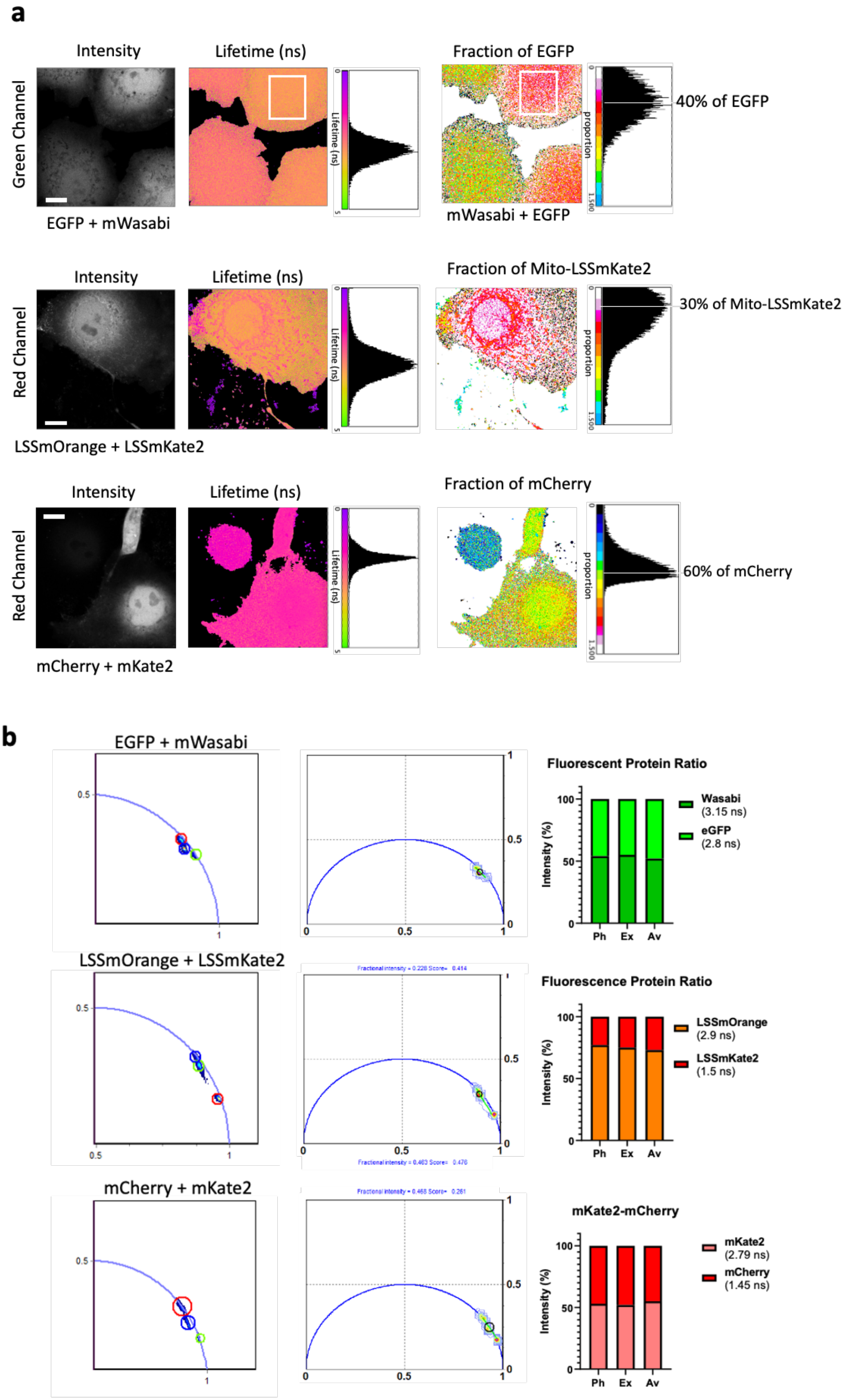
a
Intensity
Lifetime (ns)
Fraction of EGFP
Green Channel
EGFP + mWasabi
mWasabi + EGFP
40% of EGFP
Lifetime (ns)
proportion
Intensity
Lifetime (ns)
Fraction of Mito-LSSmKate2
Red Channel
LSSmOrange + LSSmKate2
30% of Mito-LSSmKate2
Intensity
Lifetime (ns)
Fraction of mCherry
Red Channel
mCherry + mKate2
60% of mCherry
b
EGFP + mWasabi
Fluorescent Protein Ratio
Intensity (%)
Wasabi (3.15 ns)
eGFP (2.8 ns)
Ph Ex Av
LSSmOrange + LSSmKate2
Fluorescence Protein Ratio
LSSmOrange (2.9 ns)
LSSmKate2 (1.5 ns)
mCherry + mKate2
mKate2-mCherry
mKate2 (2.79 ns)
mCherry (1.45 ns)

Supplementary Figure 2. Single pixel pairwise color separation for each channel. Non-fitting analytical solution as described in Methods is demonstrated for green and red channels. Intensity images of cells co-expressing either EGFP and mWasabi (green channel), LSSmOrange and LSS-mKate2 (red channel) and mCherry and mKate2 (red channel) are shown for both, the average lifetime approach (in nanoseconds, ns) (a) and the phasor analysis (b). Lifetime images (middle micrographs), pixel histograms and the calculated fraction of fluorescent protein pixel-by-pixel (right micrographs) are also shown in the second and third rows. Scale bar 2 μm. (b) The Phasor Plot and the calculation of the proportion of each species are shown in the first and second columns for the three couples (EGFP + mWasabi, LSSmOrange + LSSmKate2 and mCherry + mKate2). The third row shows the corresponding bar diagrams for the calculation of proportions employing the Phasor (Ph), the fitting approach (Ex) and the average lifetime (Av). Source data are provided with this paper.

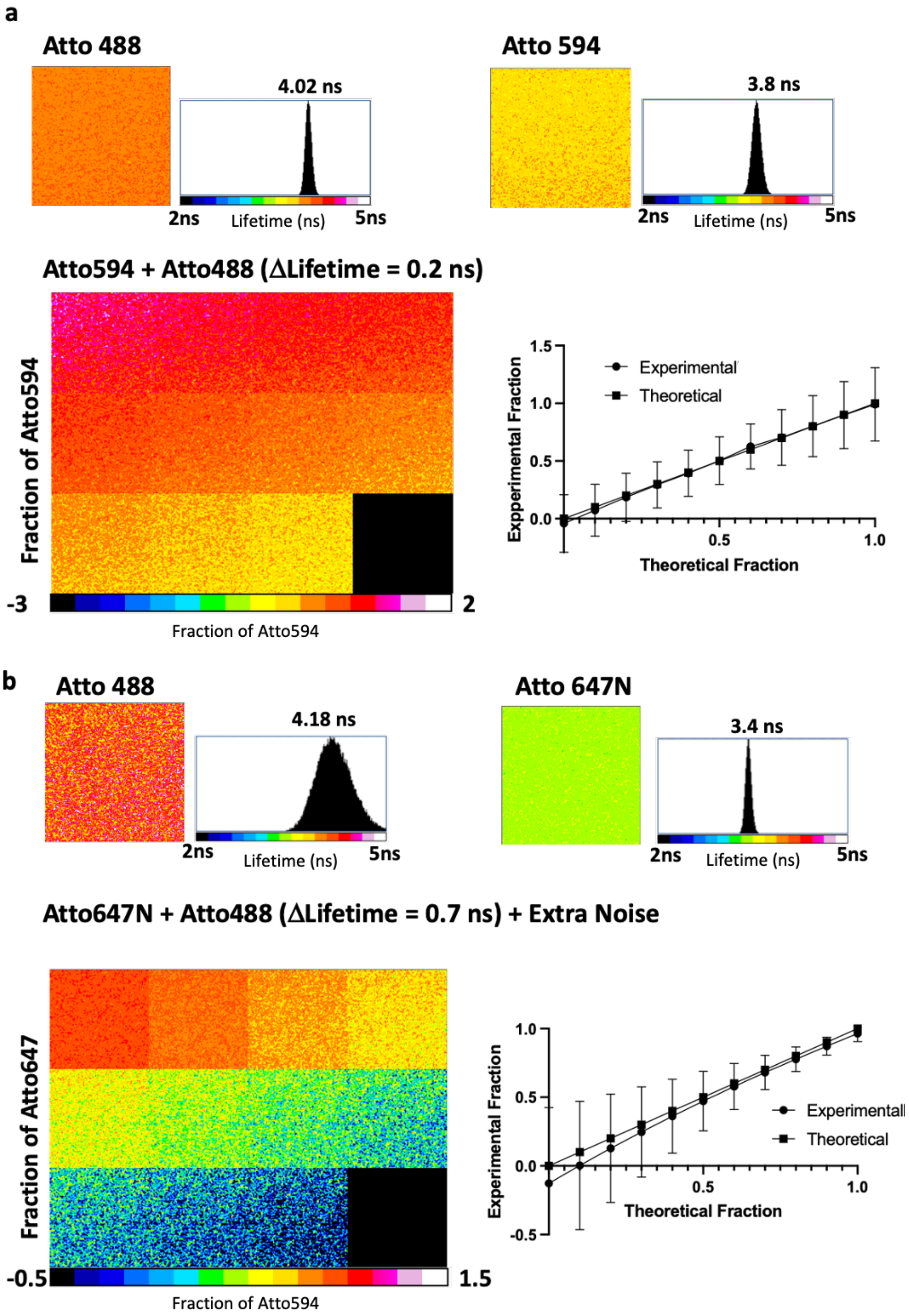
a
Atto 488
4.02 ns
2ns
Lifetime (ns)
5ns
Atto 594
3.8 ns
2ns
Lifetime (ns)
5ns
Atto594 + Atto488 (ΔLifetime = 0.2 ns)
Fraction of Atto594
-3
2
Fraction of Atto594
Expperimental Fraction
Experimental
Theoretical
Theoretical Fraction
1.5
1.0
0.5
0.0
0.5
1.0
b
Atto 488
4.18 ns
2ns
Lifetime (ns)
5ns
Atto 647N
3.4 ns
2ns
Lifetime (ns)
5ns
Atto647N + Atto488 (ΔLifetime = 0.7 ns) + Extra Noise
Fraction of Atto647
-0.5
1.5
Fraction of Atto594
Experimental Fraction
Experimentall
Theoretical
Theoretical Fraction
1.0
0.5
0.0
-0.5
0.5
1.0

Supplementary Figure 3. Mixing of ideal two species solutions to evaluate the error of the average lifetime unmixing approach. Two fluorophores with close and know lifetimes Atto488 and Atto594 (4 nanoseconds (ns) and 3.8 ns) were mixed with known proportions to evaluate the error of the unmixing approach developed in material and methods. For each solution the right panel shows the ideal result (theoretical, squares) and the calculated one (experimental, circles). (b) The addition of noise in the acquisition of atto488 when mixed with Atto647N was also evaluated. Although the difference in lifetime of these two fluorophores is bigger than in (a) (0.6 ns) the addition of noise has a bigger impact in the calculation of each proportion (right panel). Each value in the graph corresponds to a single image and the error provided to the standard deviation across all pixels of the corresponding image with known proportions of Atto488 and Atto594 (a) or Atto488 and Att0647N. Source data are provided with this paper.

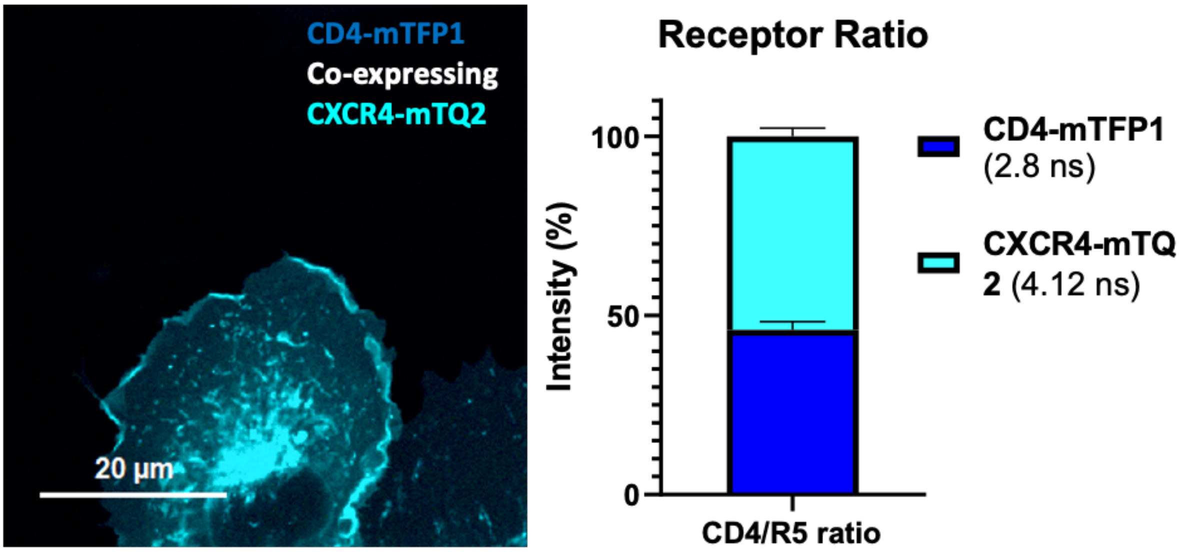

Supplementary Figure 4. Cells co-expressing CD4-mTFP1 and CXCR4-mTQ2 not engaged in the virological synapse. Live cells co-expressing CD4-mTFP1 and CXCR4-mTQ2 were analyzed to check for the overall proportion of these receptors in normal conditions. Left panel, cell co-expressing CD4-mTFP1 (dark blue) and CXCR4-mTQ2 (cyan). Right panel, a bar graph showing the proportion of expression of CD4-mTFP1 (lifetime fixed at 2.8 nanoseconds (ns)) and CXCR4-mTQ2 (lifetime fixed at 4.12ns). The bar shows an average proportion of CD4-mTFP1 of 45.9±2.3 n = 7 and CXCR4-mTQ2 of 54.1±2.3 n = 7. n denotes number of viruses examined over 3 independent experiments. Data are presented as mean values +/- SD" as appropriate. Source data are provided with this paper.

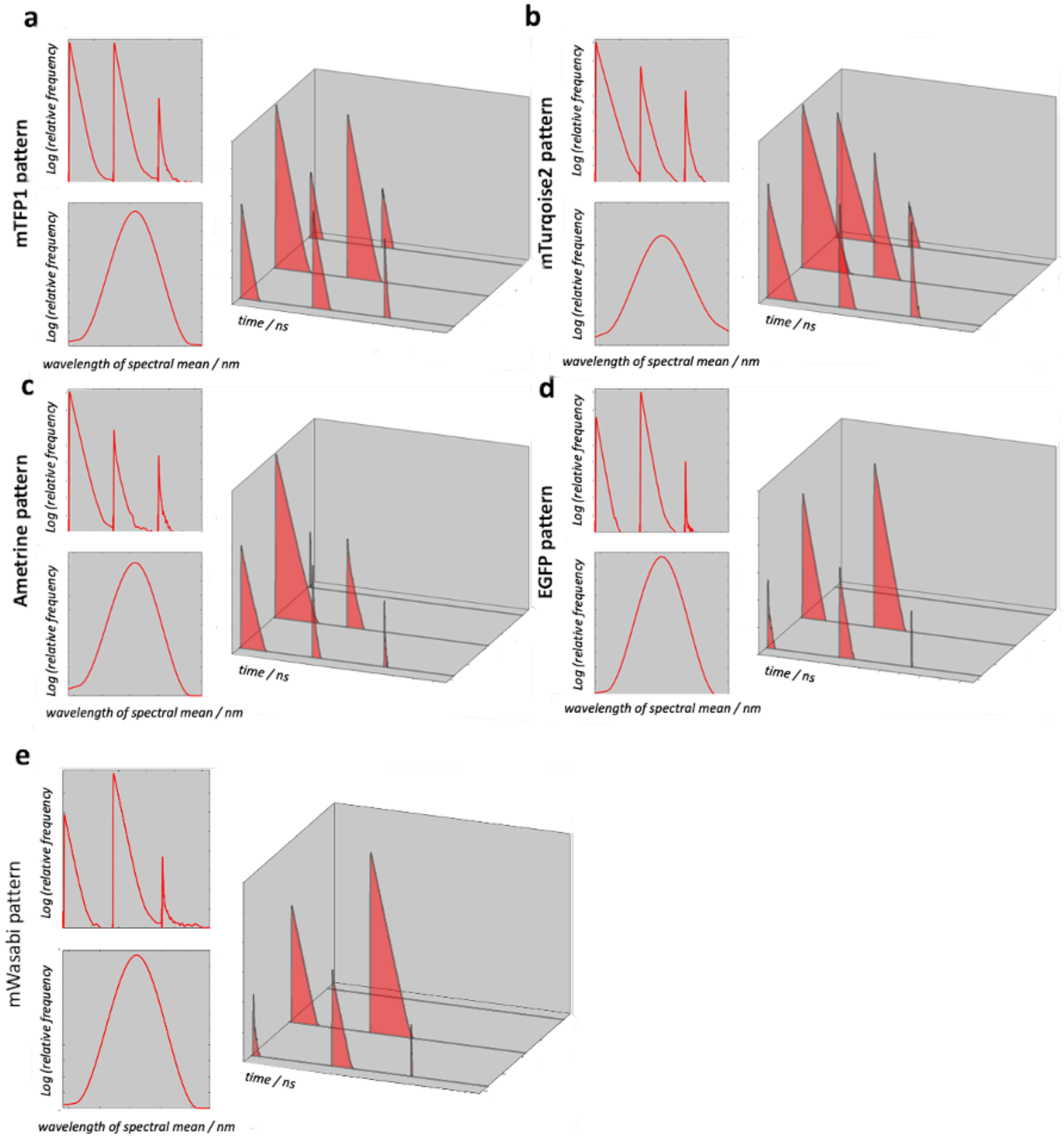

Supplementary Figure 5. Pattern Matching profiles for blue and green fluorescent proteins. The pattern matching profiles for cells expressing mTFP1 (a), mTQ2 (b), Ametrine (c), EGFP (d) and mWasabi (e) were calculated for three pulsed interleaved excitation employing three detectors with the same settings as in multicolor FLIM acquisition. The abbreviation ns denotes nanoseconds.

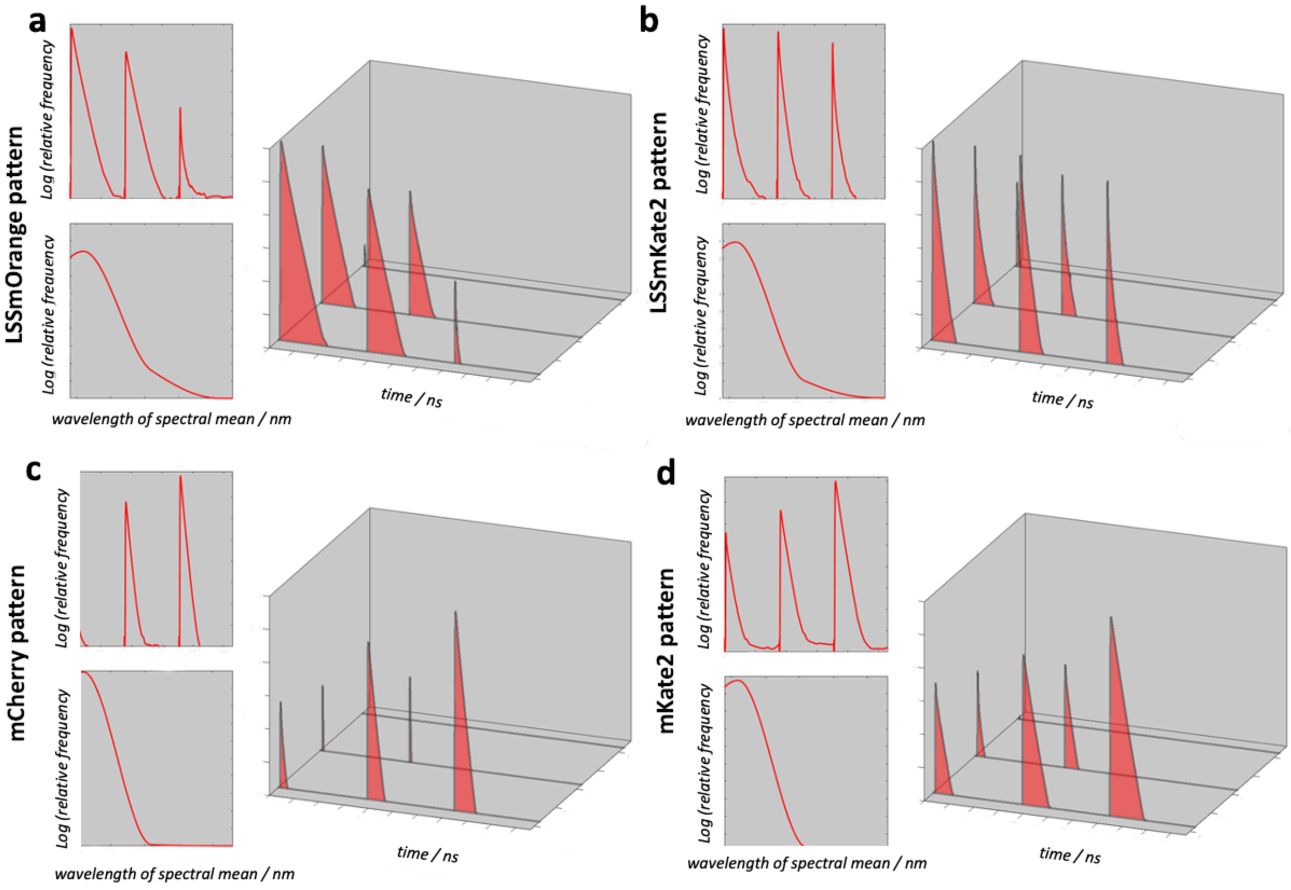

Supplementary Figure 6. Pattern Matching profiles for orange and red fluorescent proteins. The pattern matching profiles for cells expressing LSSmOrange (a), LSSmKate2 (b), mCherry (c) and mKate2 (d) were calculated for three pulsed interleaved excitation employing three detectors with the same settings as in multicolor FLIM acquisition. The abbreviation ns denotes nanoseconds.

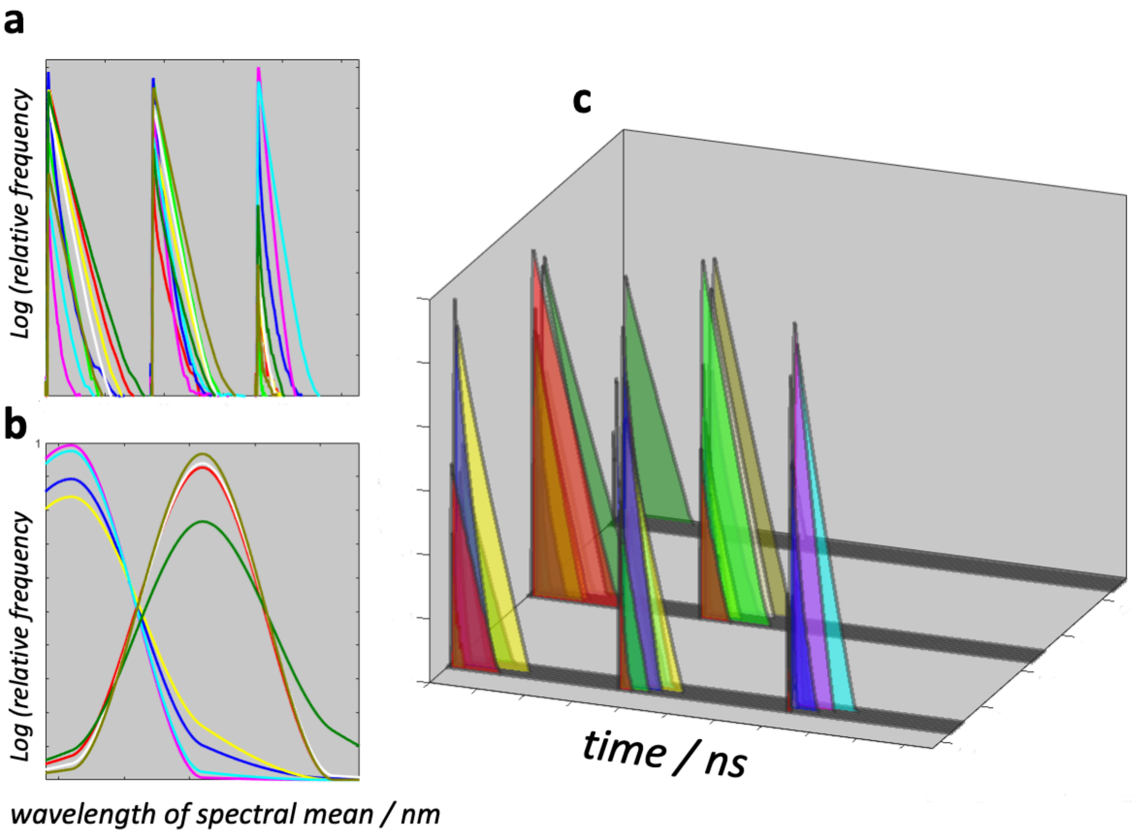

Supplementary Figure 7. Pattern Matching profiles for all fluorescent proteins. The pattern matching profiles for all cells expressing mTFP1, mTQ2, Ametrine, EGFP, mWasabi LSSmOrange, LSSmKate2, mCherry and mKate2 were calculated for three pulsed interleaved excitation employing three detectors with the same settings as in multicolor FLIM acquisition. The different decay patterns are shown in panel (a) together with the spectral profile (panel b) and the decay pattern as they were detected for each of the three channels (panel c); where each of the three detectors are shown in the z axis noted with three parallel black bold lines. The abbreviation ns denotes nanoseconds.

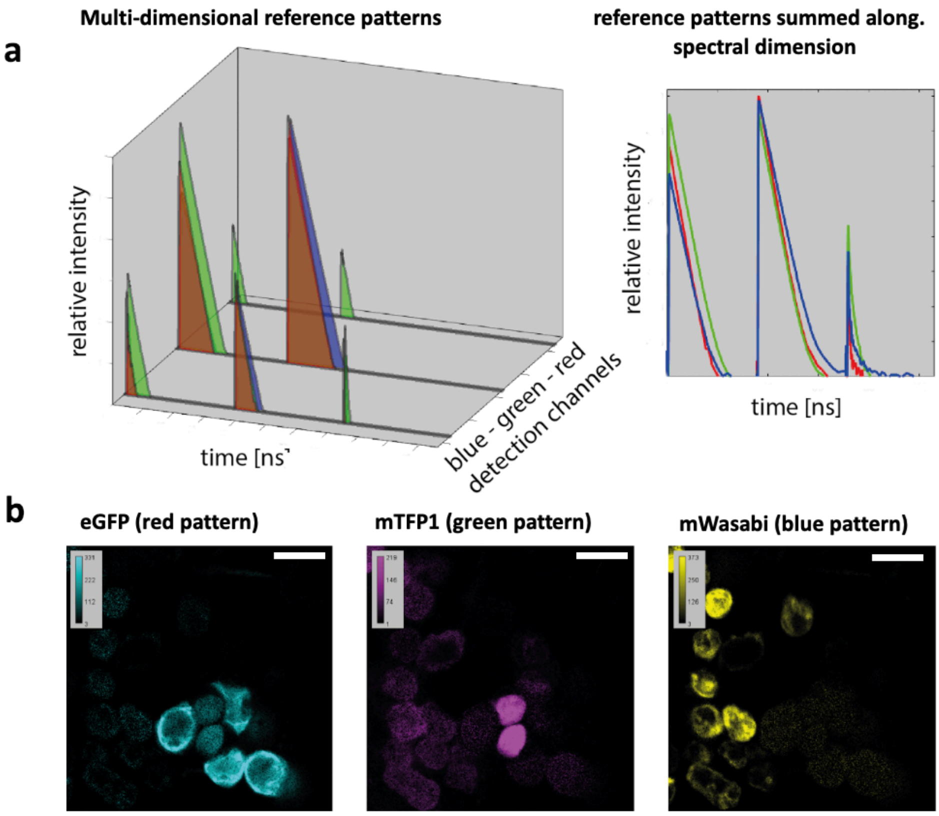

Supplementary Figure 8. Pattern Matching profiles for eGFP, mTFP1 and mWasabi. (a) Crosstalk signature between eGFP, mTFP1 and mWasabi (spectral, top panels) and pixel by pixel (bottom panels) result of applying patten matching for these three fluorescent proteins (b): eGFP (cyan micrograph), mTFP1 (purple micrograph) and mWasabi (yellow micrograph). Scale bar 5 µm.

Quadruple labelled virus (t = 90 min)

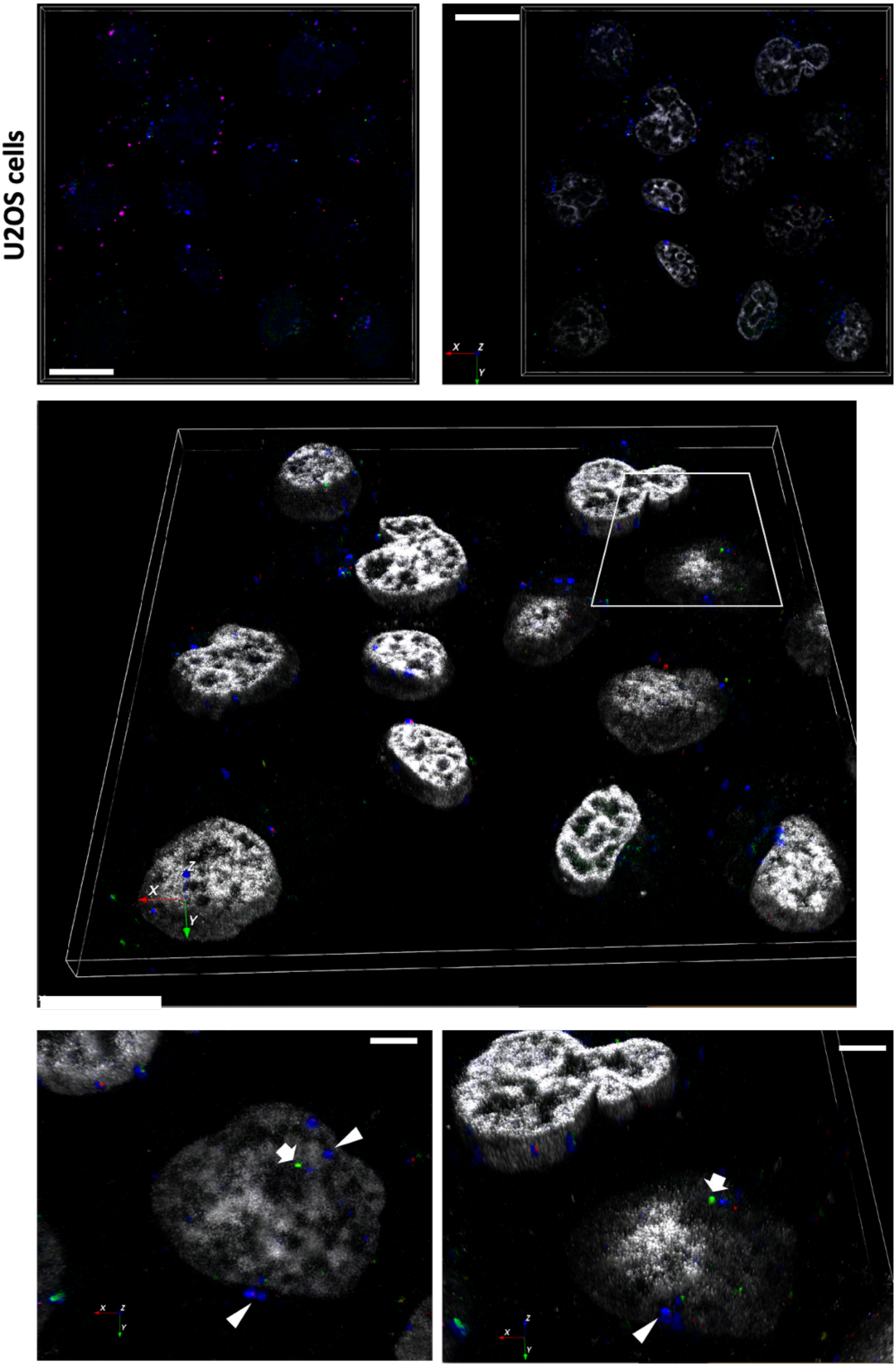

Supplementary Figure 9. Three dimensional confocal imaging of quadruple viruses exponsed to U2OS. (a) U2OS cells were exposed to quadruple labeled viruses, stained in the nucleus with DAPI and imaged at (t = 90 min). Merged images coming from 6 different channels are depicted: (transmission, grey, DAPI blue, Gag-mCherry red, CA-GFP green, Vpr-mTQ2, cyan and DiD, yellow) with and without DAPI (top panels). A three-dimensional representation is shown including the DAPI channel (grey color, midle panel). The white square is zoomed it to point at sub-viral particles within the nucleus (white arrows point at Ca-eGFP, green dots) and triangles point at Vpr-mTQ2, blue dots). These particles were only seen at t = 90 min (bottom panels). Scale bar 2 μm

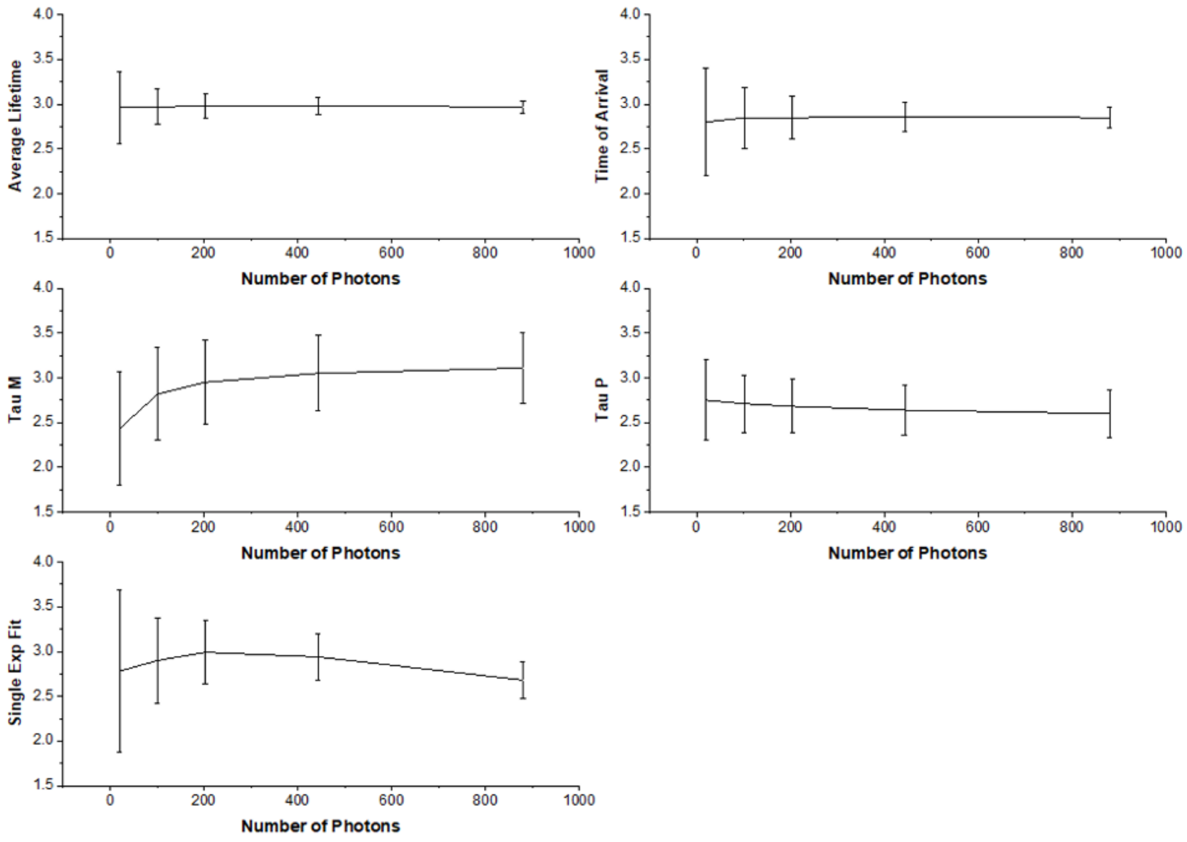

Supplementary Figure 10. Impact of the number of photons on lifetime calculation in live cells. Different lifetime analyses were tested comprising both non-fitting (top panels), phasor (middle panels) and fitting (bottom left panel) approaches. The calculation of both the average lifetime and the photon arrival time are very stable until 20-50 photons per pixel; where the lifetimes are still reliable, but their error (standard deviation) starts to increase. Single exponential approaches turned out to be much dependent on the photon budget. All calculations were made with the same sample consisting on a TZM-bl cell expressing CD4-mTFP1 alone. The data was calculated with single stack of images where the number of images within the stack was varied to impact the number of photons and the different lifetime calculations. Data are presented as mean values +/- SD" as appropriate. The abbreviations Tau M (tau modulation), Tau P (tau phase) and Single Exp Fit (single exponential fit). Source data are provided with this paper.

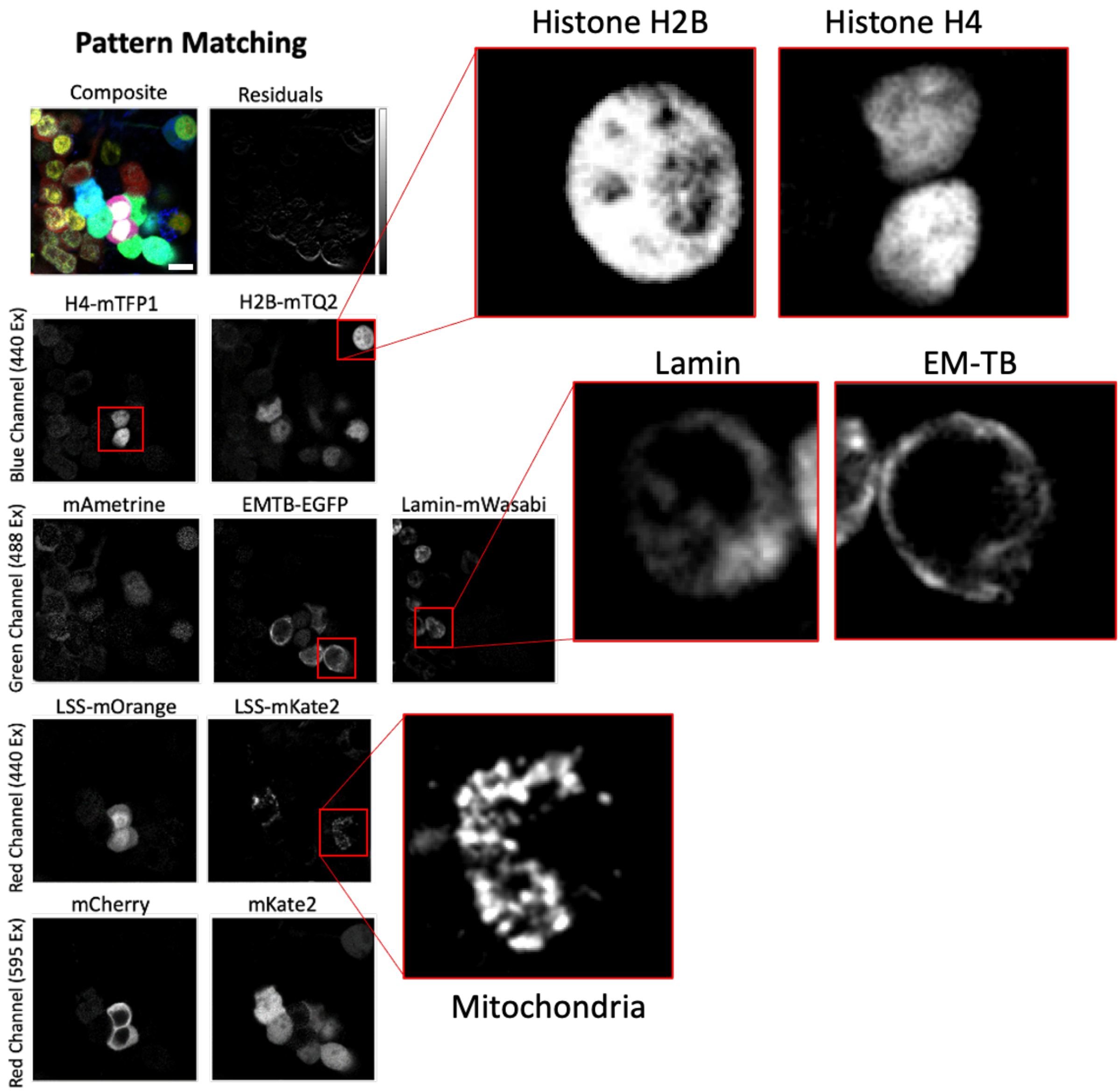

Supplementary Figure 11. Zoom ins of individual cells showing characteristics of the different sub-cellular structures. Zoomed in regions of interest (square regions) from Figure 4 are shown to highlight the sub-cellular structures resolved by pattern matching. Both histones show chromatin patterns (top panels). The tubulin (EM-TB) is also highlighted showing cables around the cytosol of HEK293T cells. Lamin is localized around the nucleus and finally the granular pattern for mitochondria are also shown (bottom panels). Scale bar. 10 µm. Abbreviation “Ex” denotes excitation.

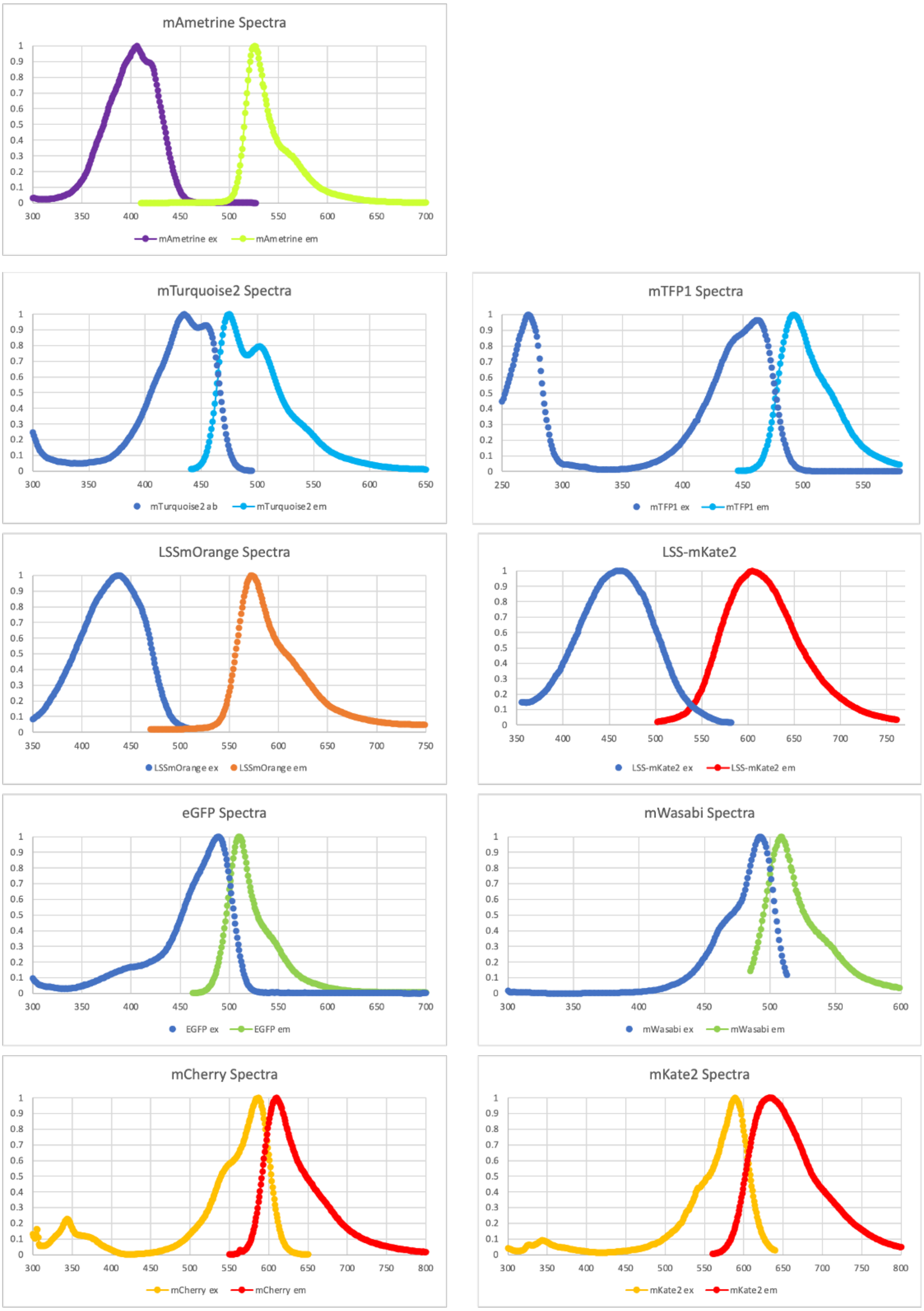

Supplementary Figure 12. Excitation and emission spectra for the 9 fluorescent proteins chosen for simultaneous multicolor FLIM. The normalized excitation spectra are shown for the nine fluorescent proteins chosen to simultaneously acquire nine colors in the multicolor FLIM approach

Supplementary Table 1. Spectral properties for individual fluorescent proteins tested when expressed in live cells.

Fluorescent Protein	Excitation (nm)	Emission (nm)	EC	QY	Brightness	pKa	Lifetime (in vitro) ns	Lifetime (COS7 cells at Room T) ns	Lifetime (COS7 cells at 37 0C) ns	Lifetime HEK293T ns (if different)	Reference (Source)
Ametrine	406	526	45,000	0.58	26.1	6	N/A	3.5	3.5	3.5	Addgene #54505
mTQ2	434	474	30,000	0.93	27.9	3.1	4	3.7	4.12	4.12	Addgene #54842
mCerulean	433	475	33,000	0.49	16.7	N/A	N/A	3	3	3	Addgene #27795
eCFP	434	477	32,500	0.4	13	4.7	3	2.4	2.4	2.4	Tramier et al., 2006 MRT
LSSmOrange	437	572	52,000	0.45	23.4	5.7	2.75		3.4	2.9	Addgene #37130
LSS-mKate2	460	605	26,000	0.17	4.42	2.7	1.4	1.5	1.5	1.4	Addgene #54795
mTFP1	462	492	64,000	0.85	54.4	4.3	3.2	2.9	2.8	2.7	Addgene #54521 and Padilla-Parra et al., 2009
NowGFP	494	502	56,700	0.76	43.09	6.2	5.1	4	4	4	Addgene #74749 and Toby/Dave
BrusLee	487	509	86,000	0.3	25.8	N/A	0.82				Mamontova et al., 2018 Scientific Rep
eGFP	488	507	55,900	0.6	33.54	6	2.6	2.69	2.7	2.7	Addgene #176015 and Padilla-Parra et al., 2009
mNeonGreen	506	517	116,000	0.8	92.8	5.7	3.1	3	3	3	From Addgene #98886
mClover3	506	518	109,000	0.78	85.02	N/A		3.2	3.2	3.2	Addgene #74236
FASTlime	481	540	45,000	0.23	N/A			1.8	1.8	1.8	Plamont et al., 2015 (PNAS)
FAST citrus	480	541	41,000	0.23	N/A			2.8	2.8	2.8	Plamont et al., 2015 (PNAS)
FASTamber	499	562	45,000	0.49	N/A			3	3	3	Plamont et al., 2015 (PNAS)
FASTcoral	518	600	39,000	0.31	N/A			3	3	3	Plamont et al., 2015 (PNAS)
mHoneyDew	487	562	17,000	0.12	2.04	4		1.8	1.8	1.8	Addgene #91760
cyRFP	529	588	48,000	0.76				3.4	3.4	3.4	Addgene #84354
sfGFP	485	510	83,300	0.65				2.68	2.68	2.68	Addgene #54579
mWassabi	493	509	70,000	0.8	56	6.5		3.1	3.1	3.1	Addgene #54743
mScarlett	569	594	100,000	0.7	70	5.3	3.9	3.5	3.4	3.4	Addgene #85042
mScarlet-H	551	592	74,000	0.2	14.8		1.3	1.4	1.3		Addgene #85043
tdTomato	554	581	138,000	0.69	95.22	4.7	N/A	2.1			Addgene #54653
TagRFP	555	584	100,000	0.48	48	3.8	2.3		1.9	1.9	Addgene #31926
mScarlet-I	569	593	104,000	0.54	56.16	5.4	3.1	3.1	2.9	2.9	Addgene 85044
mKate2	588	633	62,500	0.4	25	5.4	2.5	2.4	2.3	2.3	Shcherbakova DM et al., 2018
mCherry	587	610	72,000	0.22	15.84	4.5	1.4	1.5	1.4	1.4	Addgene #54563
mRuby2	559	600	113,000	0.38	42.94	5.3	2.5	2.2	2.2	2.2	Addgene #54768
mCardinal	604	659	87,000	0.19	16.53	5.3		1.8	1.8	1.8	Addgene #54799
mMaroon	609	657	80,000	0.11	8.8	6.2	N/A	1	1	1	Addgene #54554

Supplementary Table 2. Spectral properties for fluorescent fusion proteins tested previously.

Fluorescent Protein	Excitation (nm)	Emission (nm)	EC	QY	Brightness	pKa	Lifetime (COS7 cells at 37 0C) ns	Lifetime HEK293T ns (if different)	Reference (Source)
CCR5-mTQ2	434	474	30,000	0.93	27.9	3.1	4	4	See Methods
CXCR4-mTQ2	434	474	30,000	0.93	27.9	3.1	4	4	See Methods
Lifeact-mTQ2	434	474	30,000	0.93	27.9	3.1	4.12	4.12	Addgene #36201
H2B-mTQ2	434	474	30,000	0.93	27.9	3.1	4.12	4.12	Addgene #55557
CD4-mTFP1	462	492	64,000	0.85	54.4	4.3	2.85	2.8	See Methods
H4-mTFP1	462	492	64,000	0.85	54.4	4.3	2.9	2.9	Padilla-Parra et al., 2009 and Addgene#55491
CCR5-LSSmOrange	437	572	52,000	0.45	23.4	5.7	3.4	2.9	Addgene #110194
CXCR4-LSSmOrange	437	572	52,000	0.45	23.4	5.7	3.4	2.9	Addgene #110197
H2B-LSSmKate2	460	605	26,000	0.17	4.42	2.7	1.4	1.4	Addgene #31903
Mito-LSSmKate2	460	605	26,000	0.17	4.42	2.7	1.4	1.3	Addgene #31879
Mito-mCerulean	433	475	33,000	0.49	16.7	N/A	3	3	Addgene #55384
eGFP-Actin	488	507	55,900	0.6	33.54	6	2.7	2.7	Addgene #58149
eGFP-microtubules	488	507	55,900	0.6	33.54	6	2.6	2.6	Addgene #24327
Lifeact-eGFP	488	507	55,900	0.6	33.54	6	2.58	2.6	Addgene #58470
GPI-eGFP	488	507	55,900	0.6	33.54	6	2.6	2.6	Addgene #182866
EMTB-eGFP	488	507	55,900	0.6	33.54	6	2.6	2.6	Addgene #26741
NowGFP-CD3Z	494	502	56,700	0.76	43.09	6.2	4	4	See Methods
GFPopt-HXB2	488	507	55,900	0.6	33.54	6	2.2	2.35	Nakane et al., 2015 (JBC)
LaminB-Wassabi	493	509	70,000	0.8	56	6.5	3.1	3.1	Addgene #56507
mHoneyDew--CD3Z	487	562	17,000	0.12	2.04	4	1.8	1.8	See Methods
Actin-mScarlet	569	594	100,000	0.7	70	5.3	3.4	3.4	Addgene #85056
Lifeact-mScarlet	569	594	100,000	0.7	70	5.3	3.55	3.15	Addgene #85054
Lifeact-mRuby2	559	600	113,000	0.38	42.94	5.3	2.2	2.2	Addgene #54560
Lifeact-TagRFP	555	584	100,000	0.48	48	3.8	1.9	1.9	Addgene #54586
MitoTagRFP	555	584	100,000	0.48	48	3.8	1.9	1.9	Addgene #58023
LifeAct-mCherry	587	610	72,000	0.22	15.84	4.5	1.5	1.45	Addgene #54491
Gag-mCherry	587	610	72,000	0.22	15.84	4.5	1.5	1.2	Padilla-Parra et al., 2013
b-Actin-mCherry	587	610	72,000	0.22	15.84	4.5	1.4	1.4	Addgene #31949
Golgi-TdTomato	554	581	138,000	0.69	95.22	4.7	1.9	1.9	Addgene #58100
ER-TdTomato	554	581	138,000	0.69	95.22	4.7	1.8	1.8	Addgene #58097

Supplementary Table 3. Spectral bleedthrough for all channels corresponding to Figure4. Blue cells (below 4% crosstalk), green cells (4-15% crosstalk), yellow cells (15-40% crosstalk) and red cells (40-100%).

Pattern / Dye	Ametrine	eGFP	LSSmK2	LSSmOr	mCherry	mKate2	mTFP1	mTQ2	mWasabi
**Ametrine**	100.0	1.6	0.4	2.5	0.7	6.2	2.7	26.9	8.5
**eGFP**	1.4	100.0	1.4	4.9	1.3	26.5	7.6	5.5	1.8
**LSSmK2**	0.1	0.3	100.0	0.1	0.0	5.2	0.0	1.2	0.0
**LSSmOr**	1.5	2.8	0.7	100.0	94.3	1.9	85.1	0.2	0.0
**mCherry**	0.6	1.2	0.0	57.0	100.0	2.5	22.8	0.3	0.0
**mKate2**	9.9	90.1	57.2	6.1	12.2	100.0	1.6	47.4	0.6
**mTFP1**	1.3	2.4	0.0	62.1	17.5	0.5	100.0	0.2	13.2
**mTQ2**	32.1	12.2	9.7	0.2	0.3	41.7	0.4	100.0	0.3
**mWasabi**	3.8	0.8	0.0	0.0	0.0	0.1	5.3	0.3	100.0

Supplementary Note 1

The ImageJ macro utilized to recover the fraction for each solution/analyte in a two species system is given below:

```
// Introduce the known lifetimes for species A and B. The macro will calculate the proportion for the shortest lifetime species.


TauA = ;
TauB = ;

newImage("1", "32-bit black", 512, 512, 1);
run("Add...", "value=TauB");
rename("TauB-Image");

newImage("1", "32-bit black", 512, 512, 1);
run("Add...", "value=TauA");
rename("TauA-Image");

// Calculates the numerator

newImage("1", "32-bit black", 512, 512, 1);
run("Add...", "value=1");
rename("one");

imageCalculator("Divide create 32-bit", "tau","TauA-Image");
selectWindow("Result of tau");
rename("Tau/TauA");

imageCalculator("Subtract create", "one","Tau/TauA");
selectWindow("Result of one");
rename("Numerator");
close("one")

//Produces and image TauB/TauA and (TauB/TauA)^sqr

imageCalculator("Divide create 32-bit", "TauB-Image","TauA-Image");
selectWindow("Result of TauB-Image");
rename("TauB/TauA image");

imageCalculator("Multiply create 32-bit", "TauB/TauA image","TauB/TauA image");
selectWindow("Result of TauB/TauA image");
rename("Square TauB/TauA image");

imageCalculator("Multiply create 32-bit", "Tau/TauA","TauB/TauA image");
selectWindow("Result of Tau/TauA");
rename("product");

//Calculates denominator
```

```
selectWindow("Numerator");
imageCalculator("Subtract create 32-bit", "Numerator","Square TauB/TauA image");
selectWindow("Result of Numerator");
rename("second product");


imageCalculator("Add create 32-bit", "second product","product");
selectWindow("Result of second product");
rename("denominator");


//Calculates fB

imageCalculator("Divide create 32-bit", "Numerator","denominator");
selectWindow("Result of Numerator");
rename("fB");

//Closes windows

selectWindow("denominator");
close();
selectWindow("second product");
close();
selectWindow("Numerator");
close();
selectWindow("product");
close();
selectWindow("Square TauB/TauA image");
close();
selectWindow("TauB/TauA image");
close();
selectWindow("Tau/TauA");
close();
selectWindow("TauA-Image");
close();
selectWindow("TauB-Image");
close();
```
