# Supplementary material for: Multicolor lifetime imaging and its application to HIV-1 uptake
Source: Nat Commun. 2023 Aug 17;14:4994. doi: 10.1038/s41467-023-40731-x (PMC10435470; doi:10.1038/s41467-023-40731-x)
Supplement: Supplementary file 3 — Reporting Summary [file 41467_2023_40731_MOESM3_ESM.pdf]

Corresponding author(s): Sergi Padilla-Parra
Last updated by author(s): 2023/07/15

Reporting Summary

Nature Portfolio wishes to improve the reproducibility of the work that we publish. This form provides structure for consistency and transparency in reporting. For further information on Nature Portfolio policies, see our Editorial Policies and the Editorial Policy Checklist.

Statistics

For all statistical analyses, confirm that the following items are present in the figure legend, table legend, main text, or Methods section.

n/a	Confirmed	
☐	☒	The exact sample size (*n*) for each experimental group/condition, given as a discrete number and unit of measurement
☐	☒	A statement on whether measurements were taken from distinct samples or whether the same sample was measured repeatedly
☐	☒	The statistical test(s) used AND whether they are one- or two-sided<br>*Only common tests should be described solely by name; describe more complex techniques in the Methods section.*
☒	☐	A description of all covariates tested
☒	☐	A description of any assumptions or corrections, such as tests of normality and adjustment for multiple comparisons
☐	☒	A full description of the statistical parameters including central tendency (e.g. means) or other basic estimates (e.g. regression coefficient) AND variation (e.g. standard deviation) or associated estimates of uncertainty (e.g. confidence intervals)
☒	☐	For null hypothesis testing, the test statistic (e.g. *F*, *t*, *r*) with confidence intervals, effect sizes, degrees of freedom and *P* value noted<br>*Give P values as exact values whenever suitable.*
☒	☐	For Bayesian analysis, information on the choice of priors and Markov chain Monte Carlo settings
☒	☐	For hierarchical and complex designs, identification of the appropriate level for tests and full reporting of outcomes
☒	☐	Estimates of effect sizes (e.g. Cohen's *d*, Pearson's *r*), indicating how they were calculated

Our web collection on statistics for biologists contains articles on many of the points above.

Software and code

Policy information about availability of computer code

Data collection	Symphotime 64 2.7 (Picoquant) software was employed for data collection<br>LAS X Lifescience (Leica) was employed for data collection (confocal and FLIM images)
Data analysis	Data analysis for fitting the decays (FLIM) was carried out with Symphotime 64 2.7<br>Non-fitting data analysis was performed with FIJI (ImageJ) and the macro together with a sample is provided and available<br>Globals for Images SimFCS 2 was used for Phasor FLIM analysis<br>Icy_2.4.3 MacOS was employed for virus detection and tracking

For manuscripts utilizing custom algorithms or software that are central to the research but not yet described in published literature, software must be made available to editors and reviewers. We strongly encourage code deposition in a community repository (e.g. GitHub). See the Nature Portfolio guidelines for submitting code & software for further information.

Data

Policy information about availability of data

All manuscripts must include a data availability statement. This statement should provide the following information, where applicable:
- Accession codes, unique identifiers, or web links for publicly available datasets
- A description of any restrictions on data availability
- For clinical datasets or third party data, please ensure that the statement adheres to our policy

The data supporting the findings of this study is available at https://osf.io/gtsud/

Human research participants

Policy information about studies involving human research participants and Sex and Gender in Research.

Reporting on sex and gender	N/A
Population characteristics	N/A
Recruitment	N/A
Ethics oversight	N/A

Note that full information on the approval of the study protocol must also be provided in the manuscript.

Field-specific reporting

Please select the one below that is the best fit for your research. If you are not sure, read the appropriate sections before making your selection.

- [x] Life sciences
- [ ] Behavioural & social sciences
- [ ] Ecological, evolutionary & environmental sciences

For a reference copy of the document with all sections, see nature.com/documents/nr-reporting-summary-flat.pdf

Life sciences study design

All studies must disclose on these points even when the disclosure is negative.

Sample size	Images of at least 5 different cells where acquired per condition. Most of the times we acquired 10 different samples. No sample size calculation was performed. Sample size was chosen identifying events coming from different samples and prepared in different times (days/ weeks). To improve the quality of the analytical at least five independent images come from different observation samples.
Data exclusions	No data exclusion
Replication	Experiments where performed in different days three times (all attempts were successful)
Randomization	not relevant for this study
Blinding	Analysis of the data was performed by different indivudulas and the data put together and pooled

Reporting for specific materials, systems and methods

We require information from authors about some types of materials, experimental systems and methods used in many studies. Here, indicate whether each material, system or method listed is relevant to your study. If you are not sure if a list item applies to your research, read the appropriate section before selecting a response.

Materials & experimental systems

n/a	Involved in the study
☒	☐ Antibodies
☐	☒ Eukaryotic cell lines
☒	☐ Palaeontology and archaeology
☒	☐ Animals and other organisms
☒	☐ Clinical data
☒	☐ Dual use research of concern

Methods

n/a	Involved in the study
☒	☐ ChIP-seq
☒	☐ Flow cytometry
☒	☐ MRI-based neuroimaging

Eukaryotic cell lines

Policy information about cell lines and Sex and Gender in Research

Cell line source(s)	TZM-bl cells were obtained from the NIH AIDS repository (https://www.hivreagentprogram.org/)<br>HEK293T cells were obtained from ATCC (CRL-11268)<br>COS-7 from ATCC (CRL-1651)<br>U2OS (from ATCC, HTB-96)
Authentication	Cells were checked when received
Mycoplasma contamination	routine checks for microplasma is carried out in the lab (every 3 months). All cells were tested negative for mycoplasma
Commonly misidentified lines<br>(See ICLAC register)	TZM-bl cells contain the receptors (CD4 and CCR5 CXCR4) that allow HIV-1 particles to enter the cell and are also the gold standard for HIV-1 infection tests<br><br>No commonly misidentified cells were used in this study (to our knowledge)
